# Diagnostic Performance and Reproducibility of Chromoendoscopic Classification (FOCUS Classification: Flat‐Type Dysplasia Optical Assessment by Chromoendoscopy in Ulcerative ColitiS) for Flat‐Type Dysplasia in Ulcerative Colitis–Associated Neoplasia

**DOI:** 10.1111/den.70280

**Published:** 2026-09-08

**Authors:** Kaoru Takabayashi, Shoma Murata, Motoki Sasaki, Kurato Miyazaki, Teppei Masunaga, Kumiko Kirita, Mari Mizutani, Yusuke Yoshimatsu, Yusaku Takatori, Teppei Akimoto, Hiroki Kiyohara, Shinya Sugimoto, Shintaro Kawasaki, Yohei Mikami, Noriko Matsuura, Atsushi Nakayama, Tomohisa Sujino, Yasuhiro Ito, Kazuhiro Yamanoi, Shigeki Sekine, Yasushi Iwao, Naohisa Yahagi, Takanori Kanai, Motohiko Kato

**Affiliations:** ^1^ Center for Diagnostic and Therapeutic Endoscopy Keio University School of Medicine Tokyo Japan; ^2^ Division of Gastroenterology and Hepatology, Department of Internal Medicine Keio University School of Medicine Tokyo Japan; ^3^ Division of Research and Development for Minimally Invasive Treatment, Cancer Center Keio University School of Medicine Tokyo Japan; ^4^ Department of Multidimensional Analysis of Gastrointestinal Biology, Sakaguchi Laboratory Keio University School of Medicine Tokyo Japan; ^5^ Keio Global Research Institute Keio University Tokyo Japan; ^6^ Department of Pathology Keio University School of Medicine Tokyo Japan; ^7^ Center for Preventive Medicine Keio University School of Medicine Tokyo Japan

**Keywords:** chromoendoscopy, flat‐type dysplasia, indigo carmine, interobserver agreement, ulcerative colitis–associated neoplasia

## Abstract

**Objectives:**

Ulcerative colitis‐associated neoplasia (UCAN) often presents as flat‐type dysplasia that is difficult to characterize endoscopically. We developed a FOCUS (Flat‐type dysplasia Optical assessment by Chromoendoscopy in Ulcerative colitiS) classification and evaluated its diagnostic performance and reproducibility.

**Methods:**

This single‐center retrospective study included 33 UCAN lesions from 26 patients. Prospectively recorded FOCUS classifications at 230 biopsy sites were compared with histopathology. Chromoendoscopic patterns were categorized as Type I (“suggestive of non‐dysplasia,”), Type II (“suggestive of dysplasia with low confidence”), or Type III (“suggestive of dysplasia with high confidence”). Diagnostic performance was assessed using two prespecified thresholds: Types II/III versus Type I and Type III versus Types I/II. Eight endoscopists independently classified all images; interobserver and intraobserver agreement were assessed using Fleiss' and Cohen's *κ*, respectively.

**Results:**

The dysplasia‐bearing rate increased stepwise from Type I to Type III (Type I 15.5%, Type II 58.3%, Type III 81.8%). Using the primary threshold, sensitivity was 82.0%, and specificity 75.4% (positive likelihood ratio 3.33; negative likelihood ratio 0.24). Interobserver agreement for the three‐category classification was moderate to substantial (Fleiss' *κ* 0.629; 95% CI 0.545–0.712), and median intraobserver agreement was substantial (Cohen's *κ* 0.738).

**Conclusions:**

The FOCUS classification provides clinically meaningful risk stratification for flat‐type dysplasia in a high‐risk UCAN cohort, with acceptable interobserver and intraobserver reproducibility.

## Graphical abstract

The FOCUS classification provides simple chromoendoscopic risk stratification for flat‐type dysplasia in ulcerative colitis.

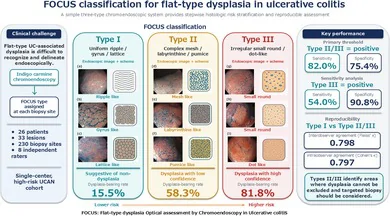

## 1 Introduction

Long‐standing ulcerative colitis (UC) is associated with an increased risk of UC‐associated neoplasia (UCAN) [1], particularly after approximately 8 years of disease duration [2, 3, 4, 5]. Because the therapeutic strategies for UCAN and sporadic neoplasia (SN) differ, distinguishing between these entities is clinically important.

UCAN frequently present as nonpolypoid lesions, and chronic mucosal inflammation leads to considerable morphological diversity, making recognition during surveillance colonoscopy challenging. Under such circumstances, one of the key issues in diagnosing UCAN is how to identify flat‐type dysplasia, which may surround or extend from an elevated lesion and represents one of the characteristic morphologies of UCAN. Several previous studies have reported that image‐enhanced endoscopy (IEE) is useful for the diagnosis of UCAN [6, 7, 8, 9, 10, 11]; however, no study has specifically focused on the endoscopic features of flat‐type dysplasia. Several reports suggested that chromoendoscopy may be effective for detecting flat‐type dysplasia [12, 13], but chromoendoscopic characteristic patterns of such lesions have not been clearly defined.

To address this gap, our group analyzed endoscopic images of flat‐type dysplasia accumulated in our institution and, in 2024, proposed a chromoendoscopic classification that described the characteristic indigo carmine dye‐spraying patterns of flat‐type dysplasia [14]. However, that classification remained somewhat oversegmented, and its clinical utility had not been formally evaluated. Therefore, in the present study, we aimed to simplify our previously proposed chromoendoscopic classification into a more practical three‐type system and to evaluate its diagnostic performance and clinical applicability for the assessment of flat‐type dysplasia.

## 2 Patients and Methods

### 2.1 Study Design and Patients

This was a single‐center retrospective analysis of prospectively recorded chromoendoscopic classifications. In routine practice, the chromoendoscopic type for each biopsy site was recorded at the time of colonoscopy in the endoscopy report; the present study retrospectively extracted these prospectively recorded classifications and compared them with histopathology. All lesions included in this study were preselected based on prior biopsy findings in which UCAN was suspected or confirmed, representing a high‐risk cohort rather than unselected surveillance cases. We included 33 lesions in 26 patients between August 2022 and August 2024. Target lesions were initially detected using white‐light imaging during routine colonoscopy, followed by chromoendoscopic evaluation.

#### 2.1.1 Definition of Simplified Chromoendoscopic Classification: FOCUS Classification (Flat‐Type Dysplasia Optical Assessment by Chromoendoscopy in Ulcerative colitiS)

**FIGURE 1:**
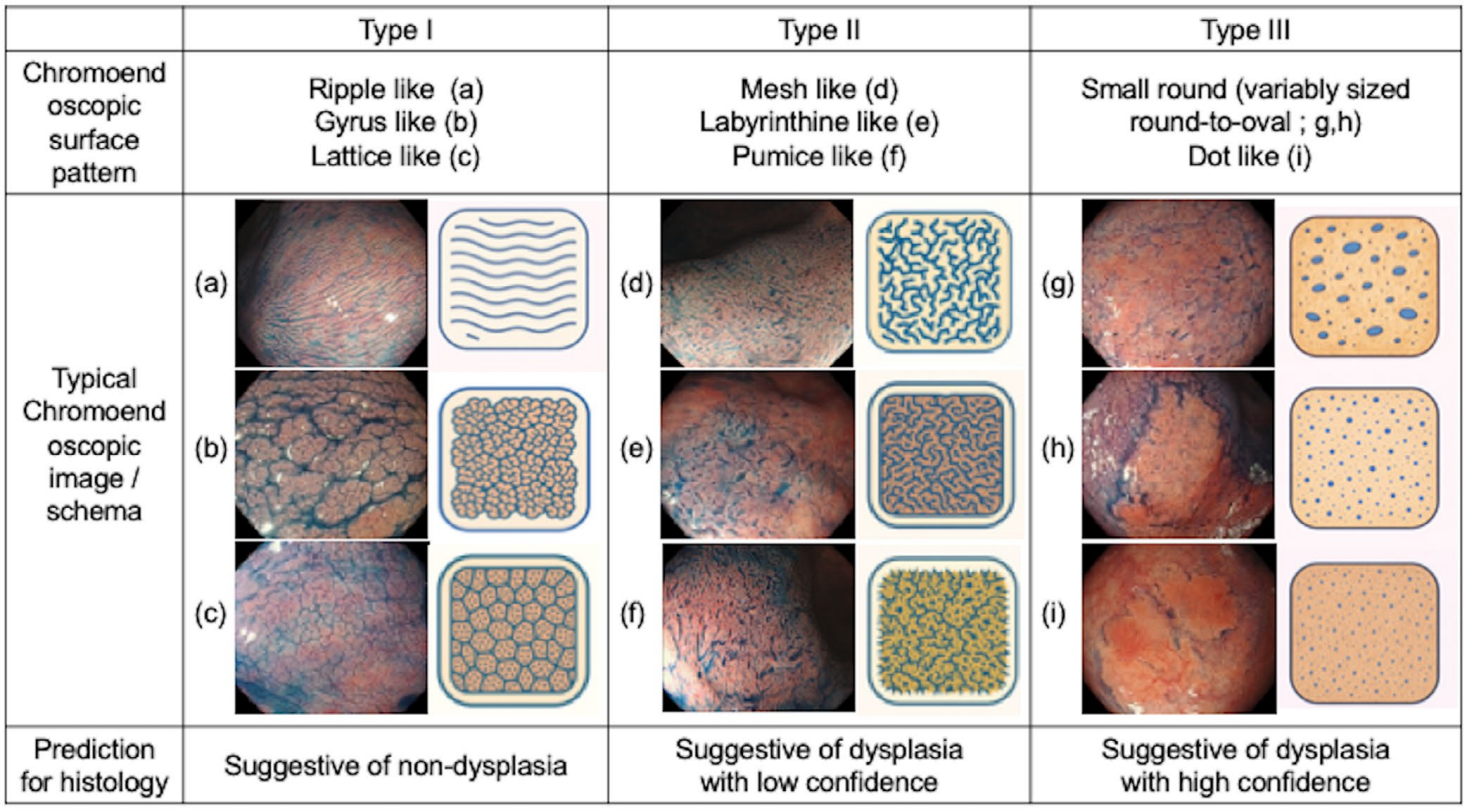
Simplified chromoendoscopic classification for flat‐type dysplasia: FOCUS classification (Flat‐type dysplasia Optical assessment by Chromoendoscopy in Ulcerative colitiS). Figure shows the simplified chromoendoscopic classification. Type I: Endoscopic findings suggestive of nondysplasia. Type I was defined as a mucosal surface showing ripple‐ (a), gyrus‐ (b), or lattice (c)‐like pattern appearing relatively uniform and homogeneous. Type II: Endoscopic findings suggestive of dysplasia with low confidence. Type II was defined as a mucosal surface showing mesh‐ (d), labyrinthine‐ (e), or pumice (f)‐like patterns appearing complex or heterogeneous in shape. Type III: Endoscopic findings suggestive of dysplasia with high confidence. Type III was defined as a mucosal surface showing a flat surface with irregular or heterogeneous small round (variably sized round‐to‐oval (g, h)) or dot (i)‐like patterns.

Figure 1 shows the chromoendoscopic classification, which is a modified version proposed at our institution for the diagnosis of flat‐type dysplasia. The mucosal surface patterns observed after indigo carmine spraying with slight magnification were classified as follows: Type I, relatively uniform ripple‐, gyrus‐, or lattice‐like patterns, suggestive of nondysplasia; Type II, complex or heterogeneous mesh‐, labyrinthine‐, or pumice‐like patterns, suggestive of dysplasia with low confidence; and Type III, irregular or heterogeneous small round (variably sized round‐to‐oval) or dot‐like patterns, suggestive of dysplasia with high confidence.

### 2.2 Definition and Evaluation of the Pathological Findings

Histologic evaluation was performed as previously described [14]. All histologic specimens were reviewed and confirmed by at least 2 experienced gastrointestinal pathologists at our hospital. The histological diagnosis of UCAN was established based on routine hematoxylin and eosin staining and p53 immunohistochemistry. The zonal expansion of p53‐positive cells, or conversely, the zonal loss of p53 expression (defined as a null‐type pattern), was considered a hallmark of clonal proliferation and served as the molecular basis for diagnosing UCAN. Diagnostic criteria for p53 included overexpression or the null‐type pattern, both manifesting as these characteristic zonal alterations, which distinguish neoplastic clones from nonneoplastic regenerative mucosa. The presence of UC‐associated inflammation and/or dysplasia in the surrounding mucosa was also considered. Additionally, we analyzed the Ki‐67 expression pattern to further differentiate UCAN from sporadic neoplasms. In sporadic neoplasms, Ki‐67 was typically localized to the luminal surface, whereas UCAN exhibited strong and diffuse expression throughout the neoplastic epithelium.

### 2.3 Evaluation of the Endoscopic Findings

Detailed chromoendoscopic examinations were performed for areas in which UCAN was suspected based on prior biopsy findings. All detailed colonoscopic and chromoendoscopic examinations were performed by two IBD specialists who were defined as a physician certified by the Japan Gastroenterological Endoscopy Society (JGES) and had performed more than 2000 colonoscopic examinations in patients with UC.

#### 2.3.1 Evaluation of Diagnostic Performance of the FOCUS Classification

Biopsy sampling followed the FOCUS classification: sites judged Type II or III were sampled for histologic confirmation, whereas negative‐control biopsies were obtained from adjacent mucosa judged Type I. Multiple biopsy sites were obtained from each lesion to assess intralesional heterogeneity. Accordingly, a single lesion could contribute multiple images and biopsy samples, each of which was analyzed independently at the site level.

To minimize missed dysplasia, we prespecified a sensitivity‐oriented primary decision rule (Type II–III = positive; Type I = negative). We also prespecified a specificity‐oriented alternative (Type III = positive; Type I–II = negative) for sensitivity analysis. We estimated the type‐specific posttest probability of dysplasia (the proportion of biopsy sites with histologic dysplasia within each Type I–III). For the classification as a whole, site‐level diagnostic performance was calculated under the prespecified decision rules, including sensitivity, specificity, and positive/negative likelihood ratios (PLR/NLR).

#### 2.3.2 Evaluation of Interobserver and Intraobserver Agreement for the FOCUS Classification

Next, interobserver and intraobserver agreement for the FOCUS classification was assessed using a retrospective review of previously acquired images. This analysis involved eight JGES board‐certified endoscopists, none of whom had participated in the development of the original chromoendoscopic classification. Prior to evaluation, all raters reviewed the reference images used to create the classification to standardize interpretation. Each rater then independently reviewed 230 chromoendoscopic still images of biopsy sites and assigned a FOCUS type to each image. Images that were judged to be of insufficient quality or not to fit any of the three categories were rated as “unclassifiable.”

Agreement was calculated for the three‐category FOCUS classification and additionally after collapsing categories according to two prespecified dichotomizations: (1) Type I versus Types II–III and (2) Types I–II versus Type III. Interobserver agreement was assessed using Fleiss' κ, whereas intraobserver agreement was assessed using Cohen's κ by having the same endoscopists reassess the same image set after an interval while blinded to their initial assessments and histopathological findings.

### 2.4 Study Procedures

Demographic and disease characteristics were collected from medical records. Colonoscopy was performed using a video colonoscope (PCF‐H290ZI or GIF‐XZ1200, Olympus Medical Systems, Tokyo, Japan). Suspected lesions identified by white‐light imaging were evaluated after spraying 0.1%–0.4% indigo carmine, and images were retrieved from the institutional endoscopy reporting system (Solemio ENDO, Olympus, Tokyo, Japan).

### 2.5 Statistical Analysis

The results are summarized as medians and interquartile range for continuous variables, and categorical variables are summarized as numbers. Sensitivity, specificity, PLR, NLR were calculated from 2 × 2 tables using standard definitions, with histopathology as the reference standard. 95% confidence intervals (CIs) for these proportions and type‐specific post‐test probability were computed using the Wilson score method. Interobserver agreement was assessed using Fleiss' *κ*, and intraobserver agreement was assessed using Cohen's *κ*. Analyses were performed for the three‐category classification and the two prespecified dichotomizations. All analyses were performed using R version 4.3.2.

### 2.6 Ethics

This study was conducted in accordance with the Declaration of Helsinki. This retrospective study was approved by the ethics committee in our institute (Approved ID 20150100).

## 3 Results

### 3.1 Patients' Characteristics

The patients' characteristics are summarized in Table 1. The median age at diagnosis was 53.0 years, and the median disease duration was 18.5 years. The male‐to‐female ratio was 14:12. Regarding disease type, 20 patients (76.9%) had the relapse and remission type, and 6 patients (23.1%) had the chronic persistent type. The disease extent was total colitis in 22 patients (84.6%) and left‐sided colitis in 4 patients (15.4%).

**TABLE 1:** Patient characteristics.

Number of patients	26
Male/female	14: 12
Age at UC onset, median (IQR), years	30.5 (15–64)
Age at tumor detection, median (IQR), years	53.0 (30–76)
Duration of disease, median (IQR), years	18.5 (11–46)
Clinical type, *n* (%)	
Relapse and remission	20 (76.9)
Chronic persistent	6 (23.1)
First attack	0 (0)
Extent of disease, *n* (%)	
Total colitis	22 (84.6)
Left‐sided colitis	4 (15.4)

### 3.2 Characteristics of Endoscopic Findings

The endoscopic characteristics are summarized in Table 2. Almost all lesions were located in the distal colon. The MES at the time of UCAN diagnosis was 0 in 26 lesions (78.8%), 1 in 7 lesions (21.2%); however, the background mucosa surrounding the lesion had an MES of 0 in all cases. Regarding macroscopic morphology, there were 12 superficial elevated lesions (36.4%), 17 flat lesions (51.5%), and 4 depressed lesions (12.1%), and all lesions contain the flat type dysplasia periphery.

**TABLE 2:** Characteristics of endoscopic findings.

Number of lesions	33
Location of neoplasia, *n* (%)	
Cecum—ascending colon	0 (0)
Transverse colon	0 (0)
Descending colon	1 (3.0)
Sigmoid colon	16 (48.5)
Rectum	16 (48.5)
Mayo endoscopic score at the time of chromoendoscopy for UCAN, *n* (%)	
MES 0	26 (78.8)
MES 1	7 (21.2)
MES 2, 3	0 (0)
Morphologic features, *n* (%)	
Superficial elevated (with flat lesion)	12 (36.4)
Flat	17 (51.5)
Depressed (with flat lesion)	4 (12.1)

### 3.3 Pathological Findings of Biopsies Obtained According to the FOCUS Classification and Type‐Specific Post‐Test Probabilities

A total of 230 biopsy samples were obtained according to the FOCUS classification (Table 3). Type‐specific posttest probabilities were as follows: within Type I, the probability of nondysplasia was 84.5% (95% CI, 76.8–90.0); within Type II, the probability of dysplasia was 58.3% (44.3–71.2); and within Type III, the probability of dysplasia was 81.8% (70.9–89.3). The FOCUS types showed a clear stepwise association with histologic findings from targeted biopsies.

**TABLE 3:** Pathological findings of biopsies obtained according to the FOCUS classification and type‐specific posttest probabilities (site level).

	Total number of biopsies	Dysplasia (+)	Dysplasia (−)	Dysplasia bearing rate % [95% CI]
		LGD (%)		
		HGD (%)		
Type I	116	18 LGD: 15 (83.3) HGD: 3 (16.7)	98	15.5 (10.0–23.2)
Type II	48	28 LGD: 28 (100) HGD: 0 (0.0)	20	58.3 (44.3–71.2)
Type III	66	54 LGD: 38 (70.4) HGD/Carcinoma: 16 (29.6)	12	81.8 (70.9–89.3)

### 3.4 Diagnostic Performance of the FOCUS Classification (Primary Decision Rule)

Using the prespecified sensitivity‐oriented threshold, the FOCUS classification showed sensitivity 82.0% (95% CI, 73.3–88.3), specificity 75.4% (95% CI, 67.3–82.0), PLR 3.33 (95% CI, 2.43–4.57) and NLR 0.24 (95% CI, 0.16–0.37) at the site level (Table 4). As a prespecified sensitivity analysis using a specificity‐oriented threshold, the corresponding values were sensitivity 54.0% (95% CI, 44.3–63.4), specificity 90.8% (95% CI, 84.6–94.6), PLR 5.85 (95% CI, 3.32–10.35), NLR 0.51 (95% CI, 0.41–0.63).

**TABLE 4:** Diagnostic performance of FOCUS classification.

Threshold	Sensitivity % [95% CI]	Specificity % [95% CI]	PLR [95% CI]	NLR [95% CI]
Type II/III = positive (primary)	82.0 (73.3–88.3)	75.4 (67.3–82.0)	3.33 (2.43–4.57)	0.24 (0.16–0.37)
Type III = positive (sensitivity analysis)	54.0 (44.3–63.4)	90.8 (84.6–94.6)	5.85 (3.32–10.35)	0.51 (0.41–0.63)

*Note:* Performance under two prespecified thresholds with Wilson 95% confidence intervals (exact binomial if small cells).

### 3.5 Interobserver and Intraobserver Agreement of FOCUS Classification

Of the 230 images (biopsy sites), at least one rater selected “unclassifiable” in 34 images (14.8%), and the remaining 196 images were classified into one of the three predefined patterns by all eight raters. Notably, images categorized as unclassifiable were considered to borderline mucosal patterns between categories. The primary interobserver agreement analyses were performed using these 196 images. For the three‐category FOCUS classification, interobserver agreement was Fleiss' *κ* = 0.629 (95% CI, 0.545–0.712), indicating moderate to substantial agreement. After category collapsing, agreement was *κ* = 0.798 (0.727–0.860) for Type I vs. Type II–III (substantial) and *κ* = 0.657 (0.541–0.758) for Type I–II vs. Type III (moderate to substantial). Intraobserver agreement was also favorable. The median Cohen's κ among the eight endoscopists was 0.738 (range, 0.631–0.963) for the three‐category classification, 0.797 (range, 0.760–1.000) for Type I versus Types II–III, and 0.786 (range, 0.515–0.945) for Types I–II versus Type III (Table 5).

**TABLE 5:** Interobserver and intraobserver agreement for the three‐category pattern classification.

	Interobserver agreement		Intraobserver agreement	
	Fleiss' *κ*	95% CI	Median Cohen's *κ*	Range of the individual *κ* coefficients
Overall (3‐category)	0.629	0.545–0.712	0.738	0.631–0.963
Type I vs. Type II/III	0.798	0.727–0.860	0.797	0.760–0.972
Type I/II vs. Type III	0.657	0.541–0.758	0.786	0.515–0.945

## 4 Discussion

In recent years, despite improved mucosal visualization with chromoendoscopy and virtual chromoendoscopy, qualitative diagnosis of UCAN has remained challenging, as highlighted in many previous reports. IEE and i‐scan can improve mucosal contrast; however, disease‐specific classification systems with validated reproducibility have not yet been established [12, 15, 16, 17, 18, 19]. In this context, the present study is, to our knowledge, the first validation study to simplify and categorize chromoendoscopic findings of flat‐type dysplasia into three types and to evaluate both diagnostic performance and interobserver agreement. The FOCUS types showed a clear stepwise association with histologic findings from targeted biopsies: Type I–classified sites were predominantly non‐dysplastic, whereas the frequency of dysplasia increased progressively from Type II to Type III. Given that HGD was detected in approximately 30% of the sites classified as Type III, whereas only LGD was detected in sites suspected to be Type II, clinicians should be aware that, when Type II and Type III patterns coexist within the same lesion, HGD may be present in the Type III area. Consequently, the posttest probability of dysplasia was low in Type I and high in Types II and III, indicating that this classification can not only distinguish dysplasia from nondysplasia but also provide an intuitive risk stratification that is easily translated into endoscopic decision‐making.

The analysis of diagnostic performance further supported the clinical utility of this system. With the primary threshold, the sensitivity and specificity for recognizing dysplasia were 82.0% and 75.4% (Table 4). Regarding Types II and III collectively as a “zone in which dysplasia cannot be excluded” may be useful for delineating lesion extent when determining the area to be resected endoscopically. In contrast, when only Type III was considered positive, sensitivity decreased to 54.0%, whereas specificity increased to 90.8%, and PLR improved to 5.85 (Table 4). These findings suggest that Type III may serve as a highly specific criterion for qualitative diagnosis. In addition, Type II may be regarded as a finding for which biopsy should be considered during surveillance because dysplasia cannot be confidently excluded. Thus, Types II/III are used for detecting dysplasia and assessing lesion extent, whereas Type III is used as a stricter indicator for qualitative diagnosis. At the same time, FOCUS Type I cannot be regarded as completely safe. In the present study, dysplasia was still diagnosed in approximately 10% of lesions classified as Type I, and all Type I areas harboring dysplasia were located adjacent to Type II or Type III regions. This finding may reflect the biological characteristics of UCAN carcinogenesis. In UCAN, dysplastic changes may arise initially in the deeper portions of the crypts, while the surface epithelium retains a regenerative or nonneoplastic appearance [20, 21, 22]. At such an early stage, the mucosal surface may not yet exhibit the structural alterations captured by the FOCUS classification, and therefore, a certain proportion of lesions harboring dysplasia in the deeper layer can be endoscopically labeled as Type I. From a clinical perspective, Type I, particularly in areas adjacent to Type II or Type III regions, should be interpreted not as an absolute exclusion of dysplasia, but rather as a finding indicating a relatively low risk. When defining the extent of a lesion, Type I may serve as a guide for selecting negative biopsy sites, provided that an adequate distance is maintained from the suspected lesion area.

With regard to qualitative diagnosis of UCAN, general colorectal classifications such as Kudo pit pattern and the Japan NBI Expert Team (JNET) classification have been applied on a trial basis. However, against the background of chronic inflammatory mucosa, neither the diagnostic accuracy for histology and depth of invasion nor the interobserver agreement has been satisfactory, and many studies have reported only fair‐to‐moderate κ values even among experts [6, 11, 23, 24, 25]. The FOCUS classification is not intended to replace these existing classifications, but rather to serve as an adjunctive tool for simple risk stratification of flat‐type dysplasia under chromoendoscopy. In the present study, agreement was highest for the clinically relevant dichotomization of Type I versus Types II/III, with an interobserver *κ* of 0.798 and a median intraobserver *κ* of 0.797. This consistent finding suggests that distinguishing Type I from Type II–III may be practical, particularly for identifying areas requiring targeted biopsy, that is, areas in which dysplasia cannot be easily excluded.

This study has several limitations. First, the lesions were preselected because UCAN had been suspected or confirmed by prior biopsy, resulting in a high‐risk cohort. Therefore, the diagnostic performance, type distribution, and predictive values may not be generalizable to unselected surveillance populations. Moreover, multiple biopsy sites from the same lesion were included in the site‐level analysis. Because observations obtained from the same lesion may be correlated, the assumption of independence may not have been fully satisfied, and intralesional clustering may have affected the estimated diagnostic performance and confidence intervals. In particular, positive predictive values and the distribution of FOCUS types are likely to differ in populations with a lower prevalence of dysplasia and a different distribution of polypoid lesions, and the usefulness of the FOCUS classification in general surveillance settings requires further investigation. Second, in approximately 15% of all biopsy‐site images, at least one rater chose “unclassifiable,” and these lesions were excluded from the primary analyses. This indicates that, under the current definitions and level of training, there remains a subset of lesions—likely influenced by image quality, active inflammation (in the present study, all evaluations were performed in segments with a background mucosa of MES 0, and the usefulness of this classification in the presence of more active inflammation has not been assessed), or borderline patterns—that cannot be readily captured by the three FOCUS types. Furthermore, because of the retrospective nature of this study, each distinct endoscopic finding may not have corresponded precisely on a one‐to‐one basis with the respective biopsy specimen, and the possibility of sampling bias cannot be excluded. This highlights the need for further refinement of the definitions and for standardized training using a larger set of representative images. Third, our evaluation was based on still images, and it remains to be determined whether similar diagnostic performance and reproducibility can be achieved during real‐time endoscopic examinations. Fourth, in this study, chromoendoscopic images were acquired by two experienced endoscopists specializing in IBD, and all evaluators were JGES board‐certified endoscopists. Therefore, the generalizability of these findings to less experienced endoscopists, other institutions, or other geographic regions should be interpreted with caution.

In conclusion, the FOCUS classification demonstrates clinically meaningful risk stratification for flat‐type dysplasia in a high‐risk setting. If validated in larger, multicenter cohorts including nonexpert endoscopists, this classification may contribute to more standardized and practical endoscopic assessment of UCAN.

## Funding

The authors have nothing to report.

## Conflicts of Interest

The authors declare no conflicts of interest.

## Data Availability

The data that support the findings of this study are available from the corresponding author upon reasonable request. The data are not publicly available due to privacy and ethical restrictions.
